# Slower progression of amyotrophic lateral sclerosis with external application of a Chinese herbal plaster–The randomized, placebo-controlled triple-blinded ALS-CHEPLA trial

**DOI:** 10.3389/fneur.2022.990802

**Published:** 2022-10-17

**Authors:** Sven Schröder, Mingzhe Wang, Dandan Sima, Joana Schröder, Xuying Zhu, Xuanlu Zheng, Lin Liu, Tingying Li, Qiudong Wang, Thomas Friedemann, Te Liu, Weidong Pan

**Affiliations:** ^1^HanseMerkur Center for Traditional Chinese Medicine, University Medical Center Hamburg-Eppendorf, Hamburg, Germany; ^2^Department of Neurology, Shuguang Hospital Affiliated to Shanghai University of Traditional Chinese Medicine, Shanghai, China; ^3^Department of Neurology, Qinghai Hospital of Traditional Chinese Medicine, Xining, Qinghai, China; ^4^Department of Integrative Neurology, Pudong Traditional Chinese Medicine Hospital, Shanghai, China; ^5^Shanghai Geriatric Institute of Chinese Medicine, Shanghai University of Traditional Chinese Medicine, Shanghai, China

**Keywords:** sporadic amyotrophic lateral sclerosis, RTC, placebo control, herbal plaster, Traditional Chinese Medicine, dysphagia

## Abstract

**Background:**

Amyotrophic lateral sclerosis (ALS) is a chronic neurodegenerative disease characterized by gradually increasing damage to the upper and lower motor neurons. However, definitive and efficacious treatment for ALS is not available, and oral intake in ALS patients with bulbar involvement is complicated due to swallowing difficulties.

**Hypothesis/purpose:**

This study investigated whether the external plaster application of the herbal composition Ji-Wu-Li efficiently slows ALS progression because prior studies obtained promising evidence with oral herbal applications.

**Study design:**

The randomized, triple-blinded study compared the efficacy, safety, and tolerability of the application of Ji-Wu-Li plaster (JWLP) with placebo plaster (PLAP).

**Methods:**

In total, 120 patients with definite ALS, clinically probable ALS, or clinically probable laboratory-supported ALS were randomized in a 1:1 ratio to receive JWLP or PLAP. Patients were treated and observed for 20 weeks. The primary outcome was the ALSFRS-R score, while the secondary outcomes were the ALS-SSIT score and weight loss.

**Results:**

The mean±SD decrease in the ALSFRS-R over 20 weeks differed by 0.84 points in a group comparison (JWLP, −4.44 ± 1.15; PLAP, −5.28 ± 1.98; *p* = 0.005). The mean increase in the ALS-SSIT over 20 weeks differed by 2.7 points in a group comparison (JWLP, 5.361.15; PLAP, 8.06 ± 1.72; *p* < 0.001). The mean weight loss over 20 weeks differed by 1.65 kg in a group comparison (JWLP, −3.98 ± 2.61; PLAP, −5.63 ± 3.17; *p* = 0.002). Local allergic dermatitis suspected as causal to the intervention occurred in 10 of 60 participants in the JWLP group and 9 of 60 participants in the PLAP group. Systemic adverse events were mild, temporary, and considered unrelated to the intervention.

**Conclusion:**

The JWLP showed clinical efficacy in the progression of ALS, as measured by the ALSFRS-R, ALS-SSIT, and weight loss in a randomized, placebo-controlled trial. Because skin reactions occurred in both groups, the covering material needs improvement. All of the *Ji Wu Li* herbal ingredients regulate multiple mechanisms of neurodegeneration in ALS. Hence, JWLP may offer a promising and safe add-on therapy for ALS, particularly in patients with bulbar involvement, but a confirmative long-term multicentre study is required.

## Introduction

Amyotrophic lateral sclerosis (ALS) is a chronic neurodegenerative disease that gradually results in increased damage to the upper and lower motor neurons (1). Its general characteristics include muscle weakness/atrophy in the oropharynx, limbs, or back muscles, dysarthria, dysphagia, eating difficulty, a choking cough, and dyspnoea (2). These features gravely affect the quality of life and may lead to respiratory failure within 3–5 years from disease onset (3, 4).

The pathophysiology of ALS is multifactorial and includes glutamate excitotoxicity (5), neuroinflammation (6), oxidative stress (7, 8), and protein aggregation (9, 10), which lead to mitochondrial dysfunction (8, 11) and apoptosis (12). Axonal transport dysfunction ultimately induces muscle atrophy (13). There is no definitive and efficacious treatment for ALS. The only established drug, riluzole, is mainly effective in the late stages of ALS (14). Moreover, due to adverse events, riluzole discontinuation is necessary for more than 20% of patients (15). Another potential treatment, edaravone, has not been approved in many countries and can only be applied intravenously; it has recently been determined to be ineffective (16). These medications are not reimbursed by insurance in many health systems worldwide.

Hence, inexpensive curative or symptomatic therapies with few adverse effects must be identified. Asian research groups have evaluated herbal medicines derived from Traditional Chinese Medicine (TCM) in animal models of ALS (17). Although the concept of ALS does not exist in TCM, a similar syndrome called “flaccidity syndrome, limpness–or atrophy syndrome” was described in the oldest medicinal book, *Huangdi Neijing*, in the context of tissue and substance loss (in TCM terms, called “Yin deficiency”) (18). Modern Chinese approaches recommend herbal drugs for ALS that are considered to have tonifying and strengthening properties (19–23).

A prior randomized clinical trial applied this concept with the oral administration of the formulation *Jia Wei Si Jun-Zi Tang* and found a slowing of the symptom progression of ALS in comparison to riluzole (23). The similar augmented herbal formula *Ji Wu Li* is a modern formula that has its basis on the classical TCM formula *Si Jun Zi* from the 12th century (24) and adds four herbs (*Astragali Radix, Rhodiola Rosea Radix, Cistanche Radix Herba, Epimedii Herba*, Table 1).

**Table 1:** Herbal ingredients of the *Ji Wu li* Plaster.

**Herbs**	**Botanical name**	**Family**	**Harvesting season**	**Processing**
*Ginseng Radix* (人蔘)	Root of Panax Jinseng C.A.Mey.	Araliceae	Any season	Dried by sunlight, its head is removed and sliced before use.
*Astragali Radix* (黃耆)	Root of Astragalus membranaceus (Fisch.) Bunge var mongholicus (Hung) Hsiao	Leguminosae	Spring and Autumn	Sliced and dried with the removal of the head and fine roots
*Cistanchis Radix Herba* (肉蓯蓉)	Fleshy stem of Cistanche deserticola Y. C. Ma	Orobanchaceae	Spring	Cleaned and cut into thick pieces without inflorescence
*Atractylodis Macrocephalae Rhizoma* (白術)	Rhizome of Atractylodes macrocephala Koidz.	Compositae	Winter	Processed by slicing and drying, it is stir-baked until a burnt color is achieved.
Poria cocos (茯苓)	Sclerotium of poria cocos (schw) Wolf	Polyporaceae	From July to September	Piled repeatedly, dried in the sun.
*Glycyrrhizae Radix* (甘草)	Root of Glycyrrhiza uraleusis Fisch	Leguminosae	Autumn	Applied crudely with honey for use after being processed by removing the root, slicing, and drying
*Rhodiola Rosea Radix* (紅景天)	Root of Rhodiola rosea L.	Crassulaceae	Autumn	Cleaned and cut into thick pieces without inflorescence
*Epimedii Herba* (淫羊藿)	Branch and leaf of Epimedium sogittaum (Sieb. Et Zucc.) Maxim.	Berberidaceae	Spring and Autumn	Dried after removal of the stem and other undesired parts. It is used roasted with sheep fat.

However, the oral intake of TCM formulations is complicated in patients with dysphagia because herbal extracts require a larger oral intake than concentrated single-component western drugs. Our ALS study team focused on external herbal medicine in the search for a better application method.

The external application of Chinese herbs has a long tradition. Since the fourth century, every TCM therapy book has included a chapter on external herbal therapy (25). Modern application forms for transdermal drug delivery include hot-melt adhesive plasters, which allow drug application directly to the skin (26). Mechanisms of external application of herbs include transdermal micro- and macro absorption, local increment of microcirculation, and adjustment of the neural-endocrine-immune network (27). In general, transdermal application reaches comparable efficacy to oral-dosage forms. However, the transdermal application has advantages because transdermal administration avoids the first-pass effect of metabolism associated with the oral route with improved bioavailability. Transdermal administration allows prolonged release, improving patient adherence and minimizing adverse effects due to lower drug peak concentrations (28, 29).

Hence, we hypothesized that the external application of herbal medicine could have similar promising effects on the progress of ALS as in the oral application form (23). Furthermore, the advantages of the external application (28, 29) could especially become relevant in ALS patients with bulbar involvement. Accordingly, we performed the present placebo-controlled, randomized, triple-blinded ALS-CHEPLA (ALS-Chinese HErbal PLAster) trial. The study aimed to compare the efficacy, safety, and tolerability of the herbal composition Ji-Wu-Li when applied as a plaster (JWLP, Table 1) and placebo plaster (PLAP) in ALS patients. The primary outcome was the Amyotrophic Lateral Sclerosis Rating Scale-Revised (ALSFRS-R) score, a self-reported instrument used to quantify the function of an individual with ALS as the disease progresses. It consists of questions covering gross motor, fine motor, oral motor, and respiratory function and shows good reliability and construct validity (30).

## Materials and methods

### Ethical approval of the study protocol

The Ethics Committee (Vote No.: KY-SHSG-2018-540) of Shuguang Hospital, affiliated with the Shanghai University of TCM, approved the study protocol. The study (trial registration number ChiCTR200037353) adhered to the Declaration of Helsinki of 1964 and its later amendments. The full trial protocol can be requested by email from the corresponding author. All patients gave their written informed consent to participate in the study and for data publication.

### Study design

This single-center, controlled, patient- and observer-blinded, parallel-group randomized trial was conducted at the Department of Neurology within Shuguang Hospital, a specialized center for motor neuron diseases. Interested individuals older than 18 with definite or probable ALS were checked for participation eligibility. Forced vital capacity (FVC) was measured at baseline. In addition, a neurologist obtained a detailed medical history and conducted a neurological examination at baseline and weeks 4, 8, 16, and 20.

### Inclusion criteria

According to revised El Escorial criteria, patients with clinically definite ALS, clinically probable ALS, or clinically probable laboratory-supported ALS were eligible for inclusion (31).

### Exclusion criteria

We excluded patients with (i) an FVC < 30%; (ii) signs of a significant psychiatric disorder and/or dementia, acute cholecystitis, or bile duct occlusion; (iii) a concomitant condition considered likely to interfere with drug adherence and outcome assessment; (iv) pregnancy; (v) short expected survival due to disease progression; and (vi) participation in other clinical trials.

### Recruitment, randomization, and masking

In total, 138 ALS patients were recruited and checked for eligibility; 120 met the inclusion criteria. Immediately after participants gave their written informed consent and before any study-related procedures were undertaken, site staff obtained a participant identification code. Eligible participants were randomly assigned following stratified randomization procedures (computerized random numbers, Microsoft Excel, 2016) at a 1:1 ratio to receive the JWLP (*n* = 60) or PLAP (*n* = 60). Randomization was stratified by sex (yes or no); there was no stratification of patients according to disease onset, age, or respiratory function.

An independent randomization center performed the randomization. They informed the study nurse about the number printed beforehand on the study medication batch, which was then connected to the participant's identification code. To achieve masking of random assignments, PLAP was matched to JWLP by appearance and packaging. Clinicians arranged patient treatment according to the participants' identification codes. The study drug was dispensed at baseline and as needed at study visits. Participants, their families, investigators, site staff, the steering committee, and anyone involved in outcome assessments were masked by these identification codes and randomization.

### Herbal and placebo preparation

The JWLP contained 32 g of herbs (*Ginseng Radix, Astragalus Radix, Cistanche deserticola Herba, Atractylodis macrocephalae Rhizoma, Poria cocos, Glycyrrhizae Radix, Rhodiola rosea Radix, and Epimedii Herba*) in a ratio of 2:6:3:2:2:2:2:2. Table 1 describes the botanical name, plant family, part of the plant, harvesting season, and processing methods. The purified raw herbs were crushed and sieved with an 80-mesh sieve. The materials were mixed with 12-g melt adhesive material [including Styrene-isoprene-styrene tertiary block copolymer (35–50%), Styrene-butadiene-styrene tertiary block copolymer (0–5%), naphthenic hydroxyl-based petroleum fractions (softener, 15–20%), C-5, cyclopentadiene and m-pentadiene (Tackifier 1, 30–40%) and esters formed by the reaction of resin acids with glycerol and pentaerythritol (Tackifier 2, 10–20%)]. To complete the plaster, we covered the self-adhesive patch with a layer of heating particles containing iron powder, salt, and activated carbon. Each plaster weighed about 100 g, including 32 g of herbs, 12 g of melt adhesive material, and 52 g of heating particles. Removal of the sealed cover paper activated the heating particles *via* contact with air; the particles were not in direct contact with the skin. The particles reached 60°C within 20 min and maintained that temperature for at least 5 h.

**Figure 1:**
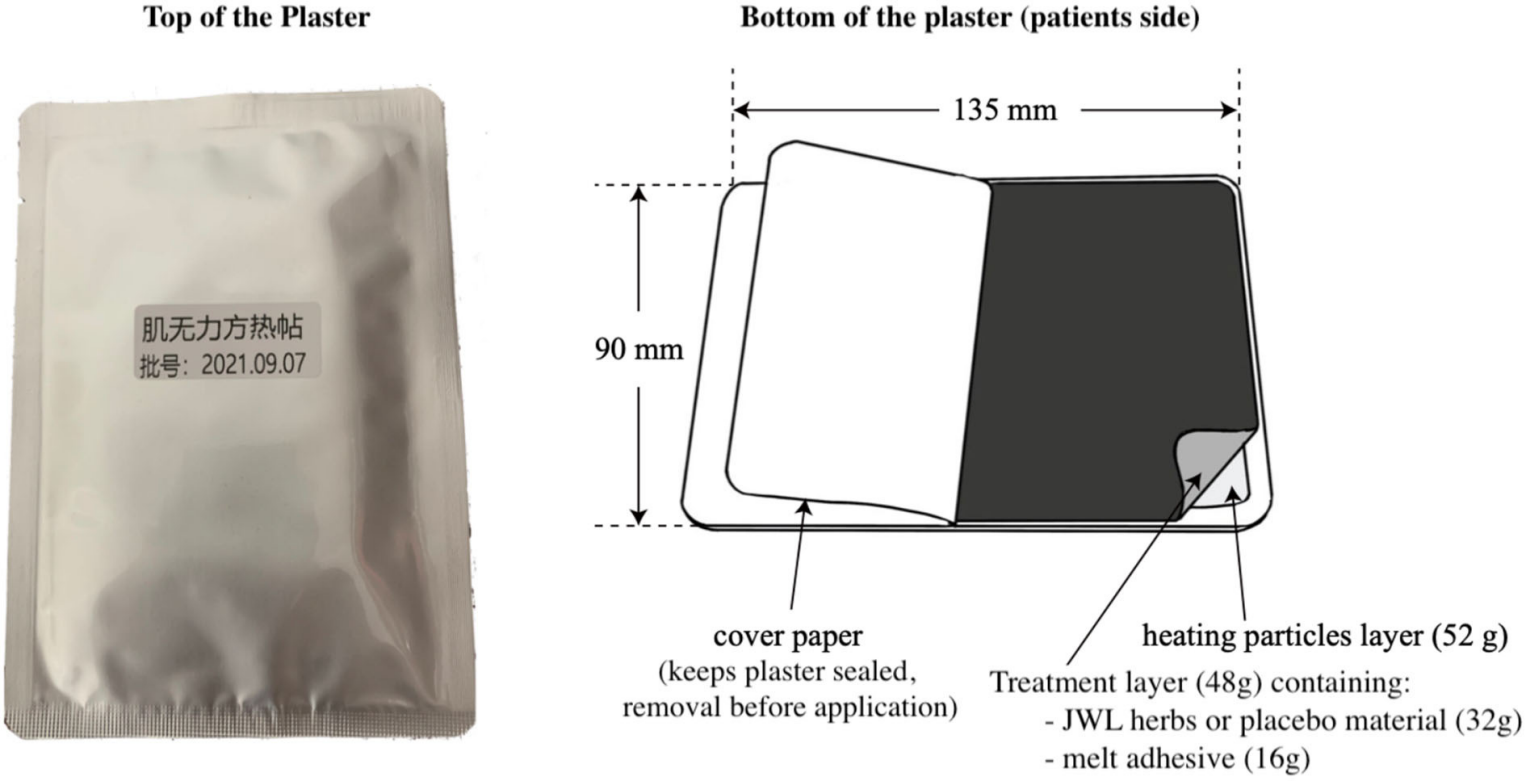
The layers of the Ji-Wu-Li plaster and the placebo.

The PLAP was prepared similarly (according to HSFA GB2760-2007, CFDA 2006, No. 120). However, a placebo (23 g soybean powder, 23 g starch, 1 g amaranth red, and 1 g carbon black pigment) replaced the herbal material. The shape, color, weight, and heat function of the PLAP were the same as those of the JWLP, and the plasters and packaging were indistinguishable. Figure 1 shows the different layers of the plaster.

### Application of the JWLP and PLAP

**Figure 2:**
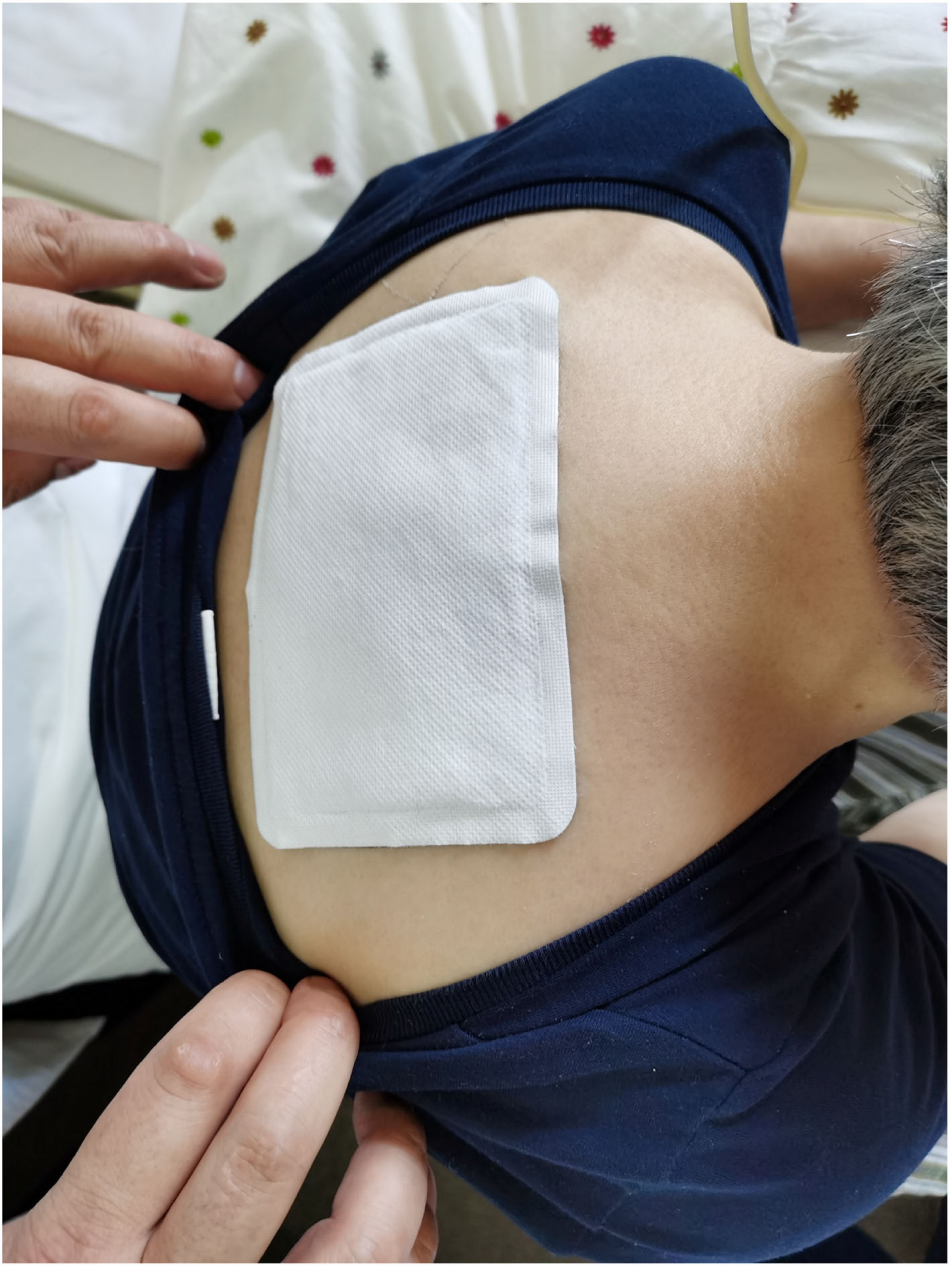
Positioning of the JWLP and the PLAP in an ALS patient.

In the JWLP and PLAP groups, the rectangular 90 × 135-mm plaster was placed on the patient's back in the midline in the depression below the spinous process of the seventh cervical vertebra. Figure 2 demonstrates the plaster position on a patient. The plaster remained in place for 6 h on 6 consecutive days, followed by 1 day of rest to reduce the skin reaction before another cycle of plaster application. The patients continued their regular medical treatment; any treatment changes had to be reported, and no change to a possibly disease-altering therapy was allowed. The description of the quality certificates of the materials of the herbs can be found in the Supplementary material.

### Primary outcome

The primary outcome was the ALSFRS-R (30). The minimum score is 0, and the maximum is 48. The lower the score, the more function is affected. A clinical neurologist assessed ALSFRS-R by interviewing the patient at baseline and weeks 4, 8, 16, and 20. The prespecified primary endpoint was 20 weeks from the baseline assessment.

### Secondary outcomes

The recently introduced ALS-SSIT (Amyotrophic Lateral Sclerosis Symptom Score in Integrative Treatments) is a clinical score reflecting the quality of life. The higher the score (a maximum score of 40), the more severe the impairment. The ALS-SSIT score has recently been validated to reflect the change in disease severity (32). A clinical neurologist assessed ALS-SSIT by interviewing the patient at baseline and weeks 4, 8, 16, and 20. A study nurse documented the participants' weight with a calibrated scale at each visit at baseline and weeks 4, 8, 16, and 20. Red blood cell count, biochemistry, kidney/liver function, and electrocardiography were assessed at baseline and trial cessation. Safety was evaluated as the prevalence and severity of adverse events and their relationship with the treatment were determined based on the results of laboratory tests, patient reports, and the judgement of the principal investigator.

### Sample size

The sample size was calculated for a two-sided *t*-test comparing the difference between two independent means using a 1:1 allocation, an alpha of 0.05, and a power of 0.8. An effect size of 0.52 was calculated based on a previous study (TCM group 3.8 ± 4.9 and control group 7.3 ± 8.2) (23). G^*^Power 3.1.9.4 was used for the sample size calculation. The results revealed that 120 patients were needed, 60 in the control group and 60 in the treatment group.

### Statistical analysis

Statistical analysis followed the intention-to-treat principle. All randomized participants were analyzed. To have an unbiased analysis, we used a complete data set for the primary analysis [JWLP (*n* = 60), PLAP (*n* = 60)]. Imputation of missing data followed an individualized decision based on predictive parameters for the missing cases. The ALSRFS-R progression rate (ΔFS' = 48–Total ALSFRS–R score at the assessment on test date/Time from onset of symptoms to assessment on test date) (33) was used to calculate the missing values by carrying the last observed progression rate forward.

Repeated-measures ANOVA was conducted to test the differences among changes in outcomes at baseline and 4, 8, 12, 16, and 20 weeks of treatment for both groups. In addition, differences at baseline and the delta from baseline to endpoint between the JWLP and PLAP were analyzed using the Student's *t*-test. *p*-value < 0.05 was considered significant. SPSS 17.0 (IBM, Armonk, NY, USA) was used for statistical analyses. Data are reported as the mean ± standard deviation.

## Results

**Figure 3:**
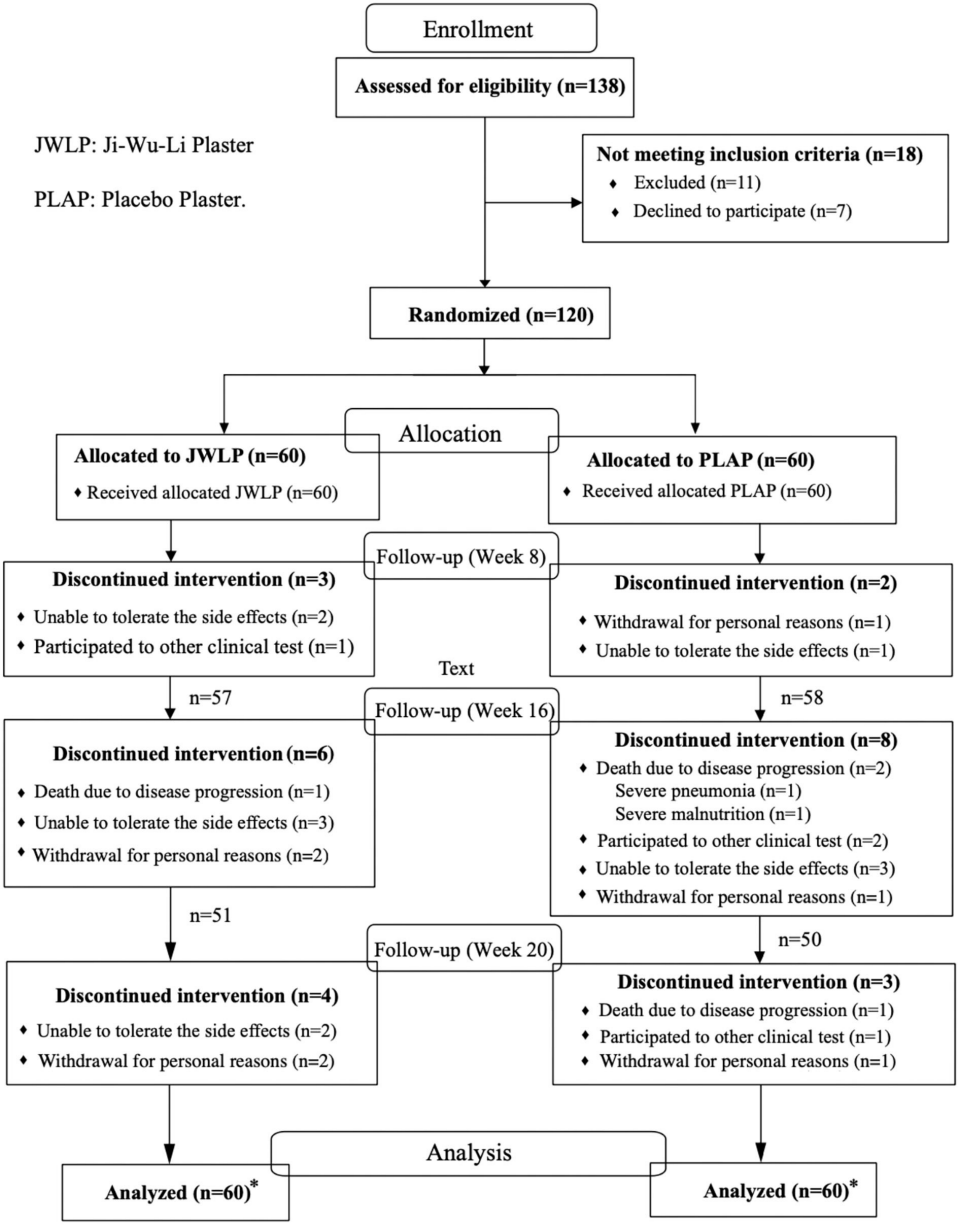
CONSORT flow diagram of the ALS-CHEPAL trial. *Intent-to-treat principle, all randomized participants were analyzed.

Figure 3 shows the CONSORT flowchart. Between July 2017 and November 2021, 138 consecutive patients were screened for eligibility, of whom 11 were excluded because they did not meet the inclusion criteria or because they met an exclusion criterion. Of the remaining 127 patients, 120 were enrolled in the trial; seven patients declined to participate.

### Basic characteristics

All ALS cases were sporadic. There were no significant differences in sex, age, body weight, onset time of morbidity, disease progression, FVC, ALSFRS-R, or ALS-SSIT between the two groups at baseline. Thirty-three participants in the JWLP group and 37 in the PLAP group had involvement of the limbs only, 13 in the JWLP group and 12 in the PLAP group had involvement of the bulbar only, and 14 in the JWLP group and 11 in the PLAP group had involvement of both the limbs and the bulbar region. Table 2 presents the basic characteristics.

**Table 2:** Characteristics of the JWLP and PLAP groups.

**Therapy**		**JWLP**	**PLAP**
Subjects	*n*	60	60
Female/male sex	*n*	24/36	24/36
Age (years)	Mean/Sd	54.9 ± 7.97	53.87 ± 8.53
Duration of the disease (month)	Mean/SD	16.45 ± 5.92	15.62 ± 5.09
ALSFRS-R (48 point scale)	Mean/SD	38.53 ± 1.99	38.50 ± 1.52
ALS-SSIT (40-point scale)	Mean/SD	20.47 ± 1.94	20.0 ± 1.76
Body weight (kg)	Mean/SD	60.45 ± 6.97	61.09 ± 6.41
Δ FS' (ALSFRS-R points/month)**	Mean/SD	0.64 ± 0.25	0.67 ± 0.24
Limbs only	*n*	33	37
Bulbar only	*n*	13	12
Limbs and bulbar	*n*	14	11
**Primary therapy**
Riluzole	*n*	27	26
Edavarone	*n*	1	1
Riluzole + Edavarone	*n*	23	24
No specific ALS therapy	*n*	9	9
FVC (%)	Mean/SD	91.8/14.9	92.2/10.9

**ALSFRS-R progression rate; FVC, Forced Vital Capacity.

### Discontinuation of the trial

Forty-seven participants in each group completed the entire 20-week observation period. Discontinuation for personal reasons occurred in four JWLP participants (two before the 16th week and two before the 20th week) and three PLAP participants (one each before the 8th, 16^th^, and 20th weeks). One JWLP patient (before the 8th week) and one PLAP patient (before the 20th week) decided to participate in another trial. One patient died before the 16th week in the JWLP group, as well as three participants in the PLAP group, two before the 16th week (one for disease progression and one for pneumonia) and one before the 20th week. Discontinuation due to non-tolerable adverse effects occurred in seven JWLP participants (two before the 8th week, three before the 16th week, and two before the 20th week) and four PLAP participants (one before the 8th week and three before the 16th week).

### Efficacy

#### Primary outcome

##### ALSFRS-R

In the JWLP group, the ALSFRS-R continuously declined from baseline (38.53 ± 1.99) to week 4 (37.53 ± 2.35), week 8 (36.34 ± 2.27), week 12 (35.72 ± 2.22), week 16 (35.12 ± 2.24), and week 20 (34.09 ± 2.19). In the PLAP group, the ALSFRS-R also continuously declined from baseline (38.50 ± 1.53) to week 4 (37.60 ± 1.78), week 8 (35.50 ± 1.66), week 12 (34.94 ± 1.68), week 16 (34.24 ± 2.17), and week 20 (33.21 ± 2.30).

**Figure 4:**
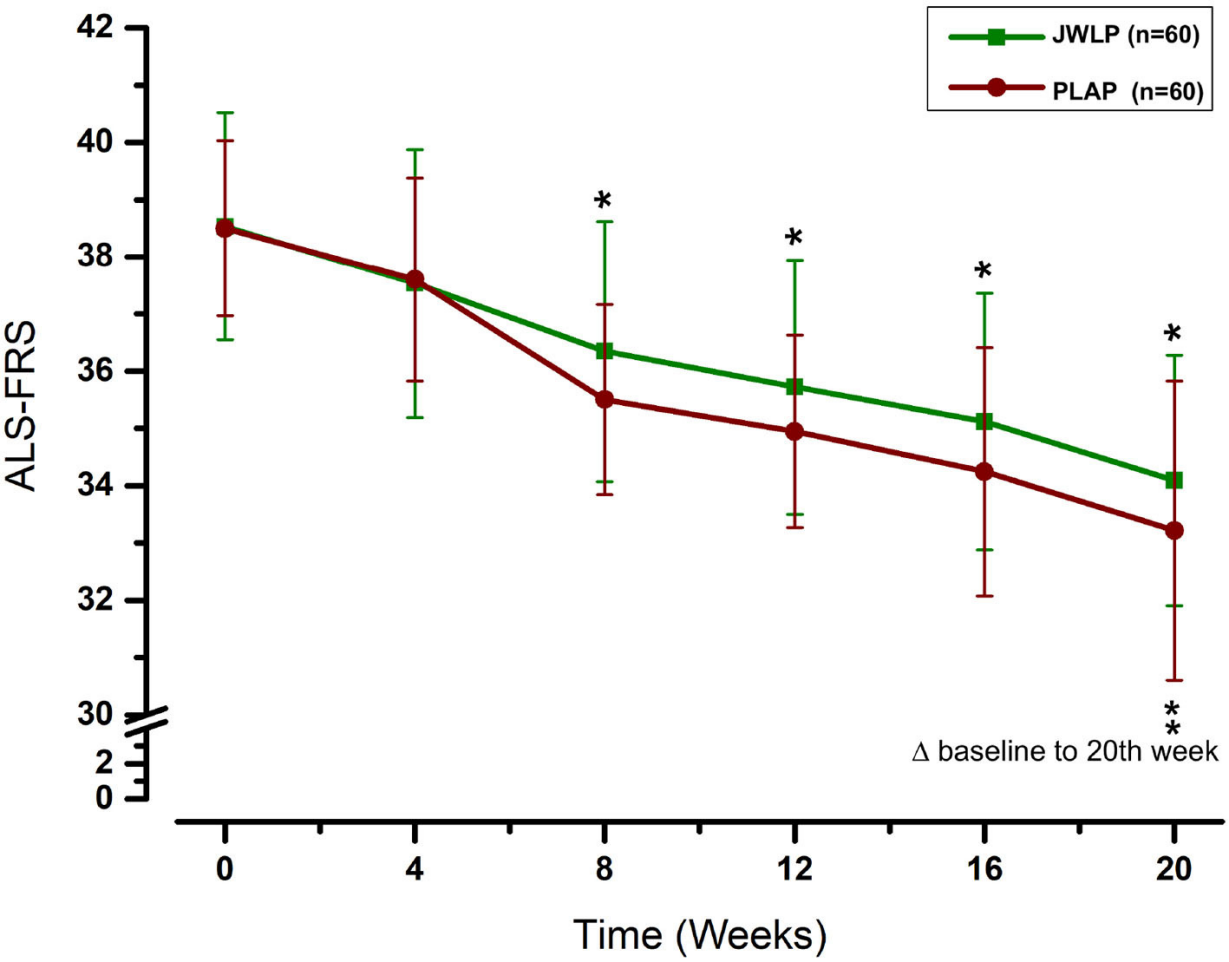
Change over time in the ALSFRS-R over the 20 week trial period. *p < 0.05, **p < 0.01.

The differences between the JWLP and PLAP were −0.03 at baseline (*t* = 0.09, 95% CI −0.71 to 0.78, *p* = 0.930), 0.07 after 4 weeks (*t* = 0.175, 95% CI −0.81 to 0.68, *p* = 0861), −0.84 after 8 weeks (*t* = 2.22, 95% CI 0.10 to 1.59, *p* = 0.027), −0.78 after 12 weeks (*t* = 2.05, 95% CI 0.03 to 1.52, *p* = 0.042), −0.87 after 16 weeks (*t* = 2.30, 95% CI 0.13 to 1.62, *p* = 0.022) and −0.87 after 20 weeks (*t* = 2.30, 95% CI 0.26 to 1.43, *p* = 0.022). Figure 4 summarizes these data. The mean decreases from baseline to the 20th week (Δbaseline to 20th week) were −4.44 ± 1.15 in the JWLP group and −5.28 ± 1.98 in the PLAP group (difference of 0.84 points, *p* = 0.005).

#### Secondary outcomes

##### ALS-SSIT

In the JWLP group, the ALS-SSIT continuously increased from baseline (20.47 ± 1.94) to week 4 (21.4 ± 2.13), week 8 (23.54 ± 1.82), week 12 (24.22 ± 1.85), week 16 (25.12 ± 1.94), and week 20 (25.83 ± 2.02). In the PLAP group, the ALS-SSIT continuously increased from baseline (20.00 ± 1.76) to week 4 (21.02 ± 1.80), week 8 (24.96 ± 1.87), week 12 (25.91 ± 1.84), week 16 (27.07 ± 2.07), and week 20 (28.06 ± 2.20). The increase in both groups was statistically significant for every 4 weeks compared to the previous score and baseline (*p* < 0.01).

**Figure 5:**
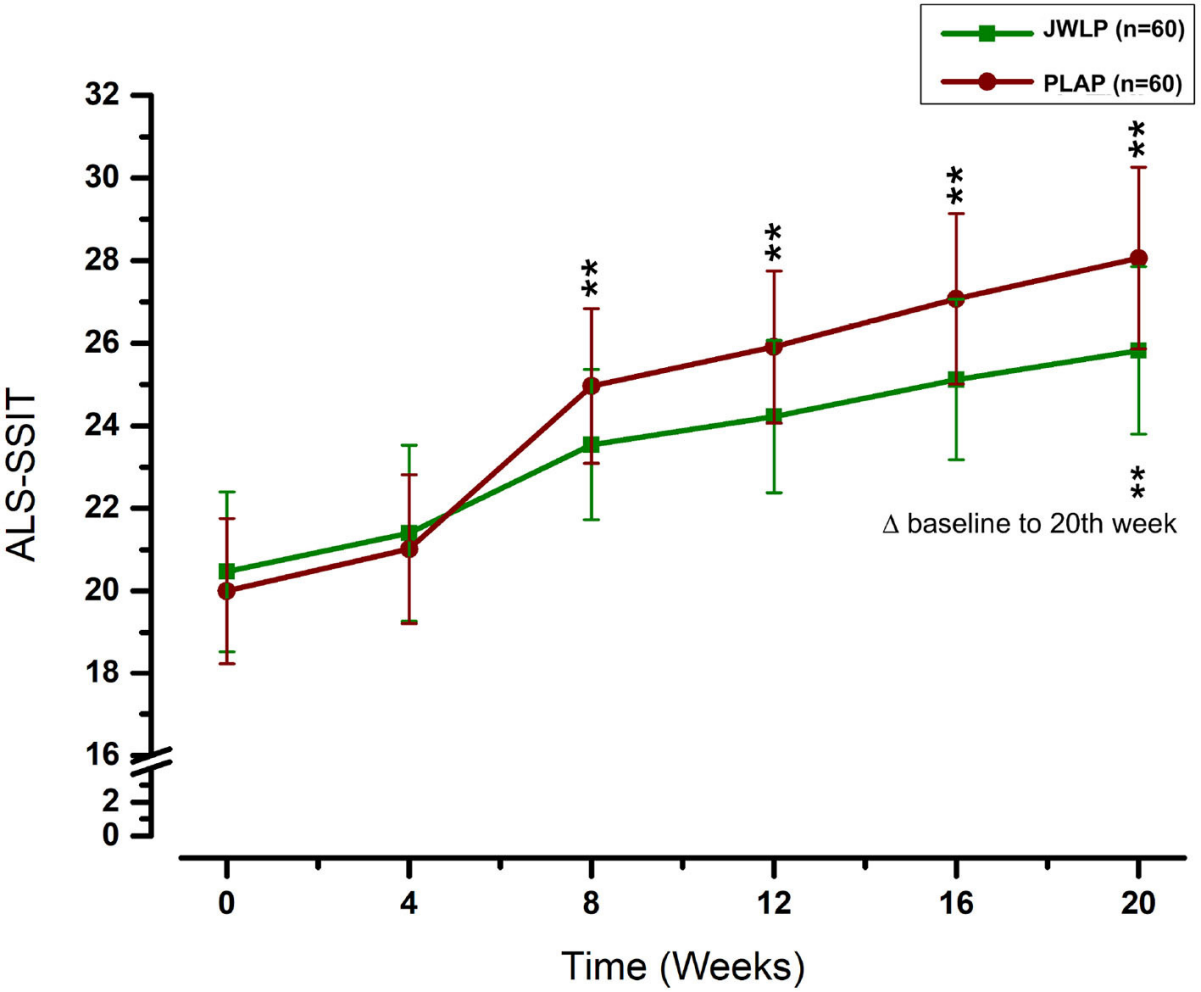
Change over time in the ALS-SITT over the 20 week trial period. **p < 0.01.

The differences between the JWLP and PLAP groups were 0.47 at baseline (*t* = 1.32, 95% CI −0.23 to 1.16, *p* = 0.188), 0.38 after 4 weeks (*t* = 1.08, 95% CI −0.31 to 1.08, *p* = 0.280), −1.42 after 8 weeks (*t* = 4.01, 95% CI −2.12 to −0.73, *p* < 0.001), −1.69 after 12 weeks (*t* = 4.77, 95% CI −2.38 to −0.99, *p* < 0.001), −1.96 after 16 weeks (*t* = 5.52, 95% CI −2.65 to −1.26, *p* < 0.001) and −2.23 after 20 weeks (*t* = 6.30, 95% CI −2.93 to −1.54, *p* < 0.001). Figure 5 summarizes these results. The increases in the ALS-SSIT within 20 weeks were 5.36 ± 1.15 points in the JWLP group and 8.06 ± 1.72 points in the PLAP group (difference of 2.7 points, *p* < 0.001).

##### Weight

**Figure 6:**
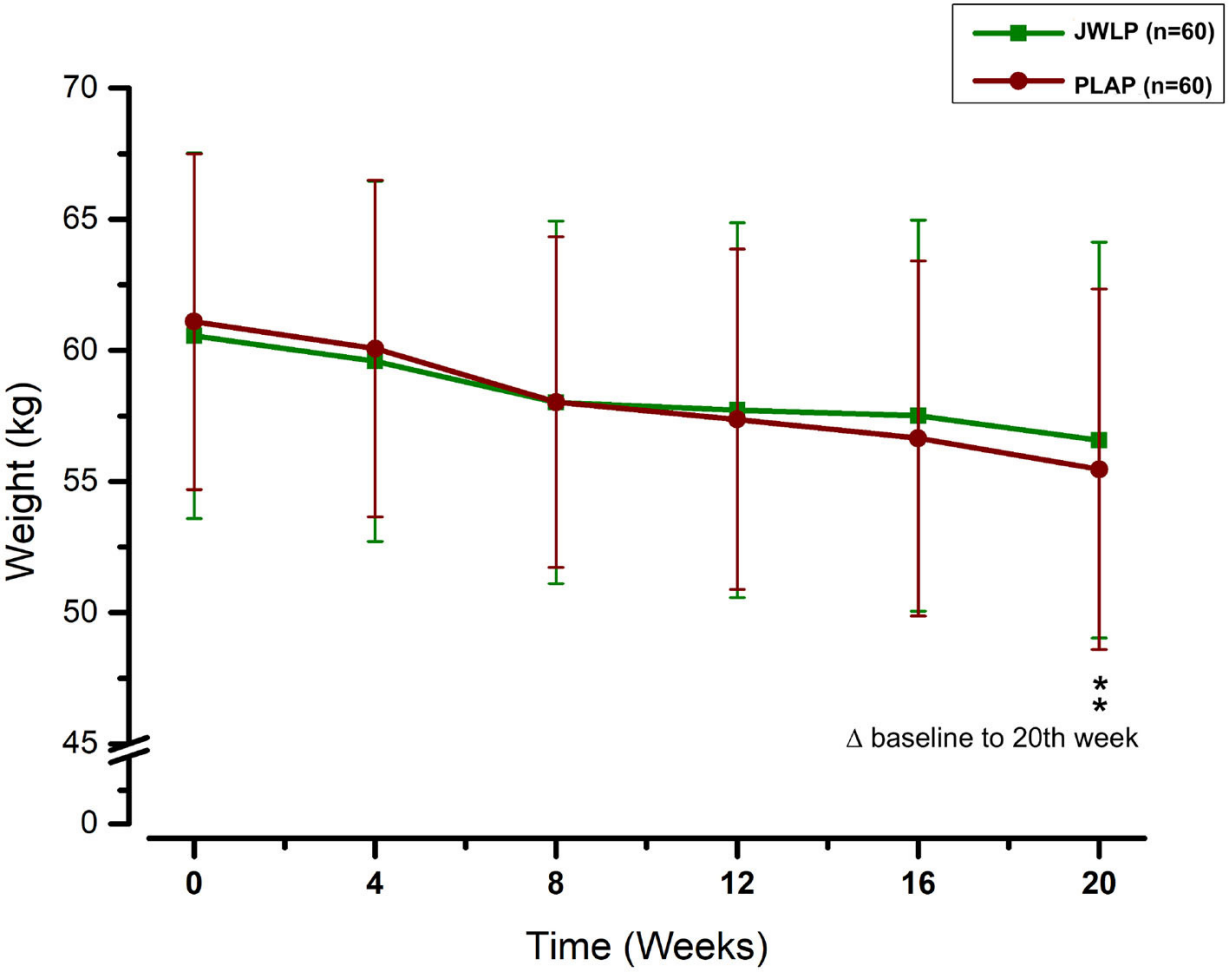
The development of the weight over the 20 week trial period. **p < 0.01.

In the JWLP group, the mean weight continuously declined from baseline (60.5 ± 6.97) to week 4 (59.58 ± 6.87), week 8 (58.01 ± 6.91), week 12 (57.72 ± 7.15), week 16 (57 ± 7.50), and week 20 (56.58 ± 7.54). In the PLAP group, the weight continuously declined from baseline (61.09 ± 6.41) to week 4 (60.07 ± 6.42), week 8 (58.03 ± 6.30), week 12 (57.37 ± 6.49), week 16 (56.64 ± 6.77), and week 20 (55.46 ± 6.86). The decrease in both groups was statistically significant for every 4 weeks compared to the previous measurement and baseline (*p* < 0.01). The difference between the JWLP and PLAP was not statistically significant at any measurement point. The mean weight decreases from baseline to week 20 were −3.98 ± 2.61 kg in the JWLP group and −5.63 ± 3.17 kg in the PLAP group (difference of 1.65 kg, *p* = 0.002). Figure 6 summarizes these findings. The results of the ALSFRS-R, ALSSITT, and weight are summarized in Table 3.

**Table 3:** ALSFRS-R, ASL-SSIT, and weight over 20 weeks.

**ASLFRS-R**		**JWLP**	**SD**	**PLAP**	**SD**	**Difference**	**95% CI**	***t*-value**	**df**	**P-value**
ASLFRS-R (48 items score)	Baseline	38.53	1.99	38.50	1.53	−0.03	−0.71–0.78	0.09	590	0.930
	4^th^ week	37.53	2.35	37.60	1.78	0.07	−0.81–0.68	0.175	590	0.861
	8^th^ week	36.34	2.27	35.50	1.66	−0.84	0.10–1.59	2.22	590	0.027
	12^th^ week	35.72	2.22	34.94	1.68	−0,78	0.03–1.52	2.05	590	0.042
	16^th^ week	35.12	2.24	34.24	2.17	−0.87	0.13–1.62	2.30	590	0.022
	20^th^ week	34.09	2.19	33.22	2.61	−0.87	0.13–1.61	2.30	590	0.022
	Δ baseline to 20^th^ week	−4.44	1.15	−5.28	1.98	−0.84	0.26–1.43	2.85	118	0.005
ALS-SSIT (40 items score)	Baseline	20.47	1.94	20.00	1.76	0.47	−0.23–1.16	1.32	590	0.188
	4^th^ week	21.40	2.13	21.02	1.80	0.38	−0.31–1.08	1.08	590	0.280
	8^th^ week	23.55	1.83	24.97	1.87	−1.42	−2.21−0.73	4.01	590	< 0.001
	12^th^ week	24.24	1.87	25.93	1.83	−1.69	−2.38–0.99	4.77	590	< 0.001
	16^th^ week	25.15	1.99	27.10	2.06	−1.96	−2.65−1.26	5.52	590	< 0.001
	20^th^ week	25.87	2.09	28.10	2.19	−2.23	−2.93−1.54	6.3	590	< 0.001
	Δ baseline to 20^th^ week	5.36	1.15	8.06	1.72	2.7	2.17–3.23	10.11	118	< 0.001
Weight (kg)	Baseline	60.56	6.97	61.09	6.41	−0.53	−2.99–1.93	0.43	590	0.670
	4^th^ week	59.58	6.87	60.07	6.42	−0.48	2.94–1.98	0.39	590	0.670
	8^th^ week	58.02	6.91	58.03	6.30	−0.01	2,47–2.45	0.01	590	0.995
	12^th^ week	57.72	7.15	57.37	6.49	0.35	−2.11–2.80	0.28	590	0.782
	16^th^ week	57.51	7.45	56.64	6.77	0.87	−1.59–3.33	0.69	590	0.489
	20^th^ week	56.58	7.54	55.46	6.87	1.11	−1.35–3.57	0.89	590	0.375
	Δ baseline to 20^th^ week	−3.98	2.61	−5.63	3.17	1.65	0.59–2.70	3.10	118	0.002

##### Safety

Allergic dermatitis under the plaster as an adverse event with a suspected causal relationship to intervention occurred in 10 of 60 JWLP group patients (16, 70%) and 9 of 60 PLAP group patients (8.33%) (df = 2, *p* = 0.098). Allergic dermatitis, though moderate and local, was not tolerable in seven JWLP participants (11.6%) and five PLAP participants (8.3%) (df = 2, *p* = 0.34) and caused discontinuation of the therapy (dropout). After cessation of the plaster treatment, all skin symptoms completely recovered and disappeared within weeks.

Temporary mild adverse events (fever, sore throat, nausea, and constipation) occurred in a minority of both groups and were considered unrelated to intervention, or a causal relationship was not assessable. No treatment-related changes in normal blood levels (red blood cells, hemoglobin, haematocrit, platelets, white blood cells, creatinine, blood urea nitrogen, y-glutamyl transferase, alanine aminotransferase, aspartate aminotransferase, and electrolytes), or alterations in the electrocardiogram related to treatment were detected.

Table 4 summarizes the adverse events.

**Table 4:** Adverse events by MedDRA preferred terms and by treatment group.

	**JWLP**		**PLAP**		**Comparison of the number of**
	**group**		**group**		**events in-between groups**
**Event type**	**Subjects *n*/60**	**Events mild/moderate/severe**	**Subjects *n*/60**	**Events mild/moderate/severe**	***P-value* *Df = 2***
**Adverse events with a suspected causal relationship to intervention**					
Local^†^ allergic dermatitis	10 (16.7%)	3/7*/0	9 (15%)	4/5*/0	0.80
**Adverse events unrelated to intervention or causal relationship not assessable**					
Temporary fever	12 (20%)	12/0/0	11 (18.3%)	11/0/0	0.18
Temporary sore throat	8 (13.3%)	8/0/0	9 (15%)	9/0/0	0.25
Temporary nausea	11 (18.3%)	11/0/0	9 (15%)	9/0/0	0.17
Temporary constipation	9 (15%)	9/0/0	7 (11.7%)	7/0/0	0.21

^†^On the plaster application site, *local skin reaction requires local symptomatic treatment with full recovery after ending of plaster application (study dropout).

## Discussion

To our knowledge, this is the first randomized, controlled, triple-blinded study of external herbal treatment for ALS. We used the ALSFRS-R score as the primary outcome, which is considered the gold standard for the staging and functional measurement of disease progression (34, 35) and comparison with newly proposed scales (36, 37). In our study, the decrease in the ALS-FRS-R was significantly lower in the JWLP group than in the PLAP group from the eighth week until the final examination after 20 weeks.

Additionally, we examined the ALS-SSIT score as a secondary outcome. The ALS-SSIT is focused on patients' quality of life and is approved due to its feasibility, reliability, validity, and sensitivity (38). Like the ALSFRS-R, the difference between the two groups was statistically significant from the eighth week onwards but became more pronounced thereafter until the endpoint. The higher measurement sensitivity of the differences and the focus on the quality of life suggests that the ALS-SSIT should be considered in future ALS studies. Furthermore, we examined weight as an observer-independent marker of cachexia. The mean weight loss for the period of 20 weeks was 1.65 kg higher in the PLAP group than in the JWLP group, which was statistically significant.

One novelty of this study was the introduction of the external application of herbs into ALS treatment. This approach is useful for patients with bulbar involvement. In general, transdermal application has comparable efficacy to oral administration. The mechanisms underlying the external application of herbs include transdermal micro-/macroabsorption, local augmentation of microcirculation, and adjustment of the neural-endocrine-immune network (27). Transdermal application has advantages because it avoids the first-pass effect of metabolism associated with the oral route, leading to improved bioavailability (39, 40).

Furthermore, it allows prolonged release, improves patient adherence, and minimizes adverse effects due to lower drug peak concentrations (29). Transdermal application may avoid gastrointestinal irritation, low absorption, and a short half-life, necessitating frequent dosing. Thus, a lower daily dose can elicit an equivalent therapeutic effect. The most significant disadvantages are the lower permeability of the skin for some herbal ingredients, the slow permeation of hydrophobic ingredients, differences from person to person and with age, and the possibility of local irritation at the application site (26). Skin reactions were the only adverse effects leading to trial cessation in this study. However, the termination rate in the JWLP group was half the rate reported for riluzole, and the adverse events were less severe (15).

Furthermore, the PLAP patients experienced similar skin symptoms. Hence, the adverse effects are partly not medication-generated but a reaction to the plaster material (melt adhesive material) or heat. Therefore, the material needs future improvement, and a periodic change in the plaster position might be an option.

The location of the plaster was chosen for practical considerations because placement in this region does not significantly hinder movement or function. Furthermore, it covers the paravertebral muscles, the trapezius muscle, and the rhomboid, which are well perfused. In addition, the plaster is positioned above reflex areas, which are traditionally considered to have toning and strengthening properties and whose stimulation leads to activation of the thoracic sympathetic trunk with sympathetic afferent neural induction of the increased perfusion of skeletal muscles. This is relevant because the sympathetic nervous system regulates skeletal muscle motor innervation and acetylcholine receptor stability. A progressive decline in sympathetic innervation is frequent in ALS with impaired adaptation to physiological stressors (41).

ALS has multifactorial mechanisms of neurodegeneration that lead to mitochondrial dysfunction (8, 11) and apoptosis (12), with a consequent dysfunction in axonal transport and muscle atrophy (13). The classical pharmacological approach that focuses on a single target of the ingredients can only have limited success (42), as shown for riluzole, which affects glutamate excitotoxicity (43), and edaravone, which affects oxidative stress alone (44)—the herbs of JWL target the pathophysiological mechanism of ALS. Taken together, these herbs target oxidative stress and neuroinflammation and potentially protect against mitochondrial dysfunction and apoptosis. Furthermore, *Ginseng Radix, Astragalus Radix, Atractylodis macrocephalae Rhizoma, Glycyrrhizae Radix, Rhodiola rosea Radix, and Epimedii Herba* can prevent glutamate excitotoxicity while *Ginseng Radix, Astragalus Radix, Glycyrrhizae Radix, and Rhodiola rosea Radix* can ameliorate skeletal muscle atrophy (Supplementary Figure 1 summarizes these findings, while Supplementary Tables 1a,b, describe the herbs, their constituents and their mechanisms of action on the targets). Overall, the ingredients of every single herb of JWL have effects on almost all known mechanisms of ALS (45–58). Hence, JWL is a promising combination of herbs that counteracts multiple mechanisms of ALS.

The present study has certain limitations. First, the work was conducted at a single center. A multicentre study is advisable for confirmation. In addition, the study evaluated only 20 weeks. Future studies should confirm the effects in a long-term clinical trial.

Furthermore, the therapeutic concept is based on traditional experience with external applications and herbal studies with oral medications. However, there is limited knowledge on the absorption rate of single ingredients or the pharmacokinetics and pharmacodynamics. Hence, targeted selective animal and human studies are mandatory to substantiate the clinical use of the JWLP in ALS patients.

Nevertheless, while there are not enough established and effective therapies for ALS, there is sufficient knowledge of the toxicology and pharmacovigilance of the single herbs of JWL. Furthermore, their use is well-established, and relevant systemic adverse effects did not occur during this study. Hence, combining TCM formulations with western medicine is an encouraging way to help alleviate symptoms and delay ALS progression.

## Conclusions

The JWLP showed clinical efficacy in a randomized, controlled, placebo-controlled trial, measured by the ALSFRS-R, ALS-SSIT, and weight loss. The study revealed no systemic adverse effects. Because skin reactions occurred in the verum and placebo groups, the covering material needs improvement. Hence, JWLP offers a promising add-on therapy for ALS, particularly in patients with bulbar involvement.

## Data availability statement

The original contributions presented in the study are included in the article/Supplementary material, further inquiries can be directed to the corresponding authors.

## Ethics statement

The studies involving human participants were reviewed and approved by Ethics Committee of Shuguang Hospital Affiliated with the Shanghai University of TCM. The patients/participants provided their written informed consent to participate in this study. Written informed consent was obtained from the individual(s) for the publication of any potentially identifiable images or data included in this article.

## Author contributions

WP, TLiu, TF, and SS conceived and designed the study and supervised the experiments. MW, DS, JS, XZhe, LL, TLi, and XZhu performed the trial, data collection, literature research, and data analysis. QW and TF performed and controlled the statistical analysis, WP and SS drafted the manuscript. All data were generated in-house and no paper mill was used. All authors corrected the draft manuscript, agreed to be accountable for all aspects of the work, ensuring integrity, and accuracy.

## Funding

The present study was supported by a grant from the National Natural Science Foundation of China (81373619) and the Clinical Research Plan of Shanghai Shenkang Hospital Development Center (SHDC2020CR2027B). Funding was also received from the Shanghai Municipal Health and Family Planning Commission (ZY3-CCCX-3-3030).

## Publisher's note

All claims expressed in this article are solely those of the authors and do not necessarily represent those of their affiliated organizations, or those of the publisher, the editors and the reviewers. Any product that may be evaluated in this article, or claim that may be made by its manufacturer, is not guaranteed or endorsed by the publisher.

## Supplementary material

Supplementary Figure 1 Pharmacological treatment targets in relation to ALS disease mechanism.

Supplementary Table 1a Antioxidative and anti-inflammatory activities of the main constituents of *Ji Wu Li*.

Supplementary Table 1b Neuroprotective and Glutamate excitotoxicity- and muscle atrophy attenuating effects of *Ji Wu Li* constituents. ABTS, 2,2′-Azino-bis(3-Ethylbenzothiazoline-6-Sulfonic Acid); AChE, Acetylcholinesterase; AIF, apoptosis-inducing factor; AMPK, AMP-activated protein kinase; Bax, Bcl-2-associated X protein; Bcl-2, B-cell lymphoma 2; CARM1, co-activator-associated arginine methyltransferase 1; CAT, Catalase; COX2, Cyclooxygenase-2; DAPK1, Death Associated Protein Kinase 1; DNA, deoxyribonucleic acid; DPPH, 2,2-Diphenyl-1-picrylhydrazyl; ERK, Extracellular-signal Regulated Kinases; GLT-1, Glutamate transporter-1; GPx, Glutathione peroxidase; GSH, Glutathion in its reduced form; GSH-PX, Glutathionperoxidase; GSK3β, Glycogen synthase kinase 3 beta; HSP-16.2, Heat-shock-protein 16.2; Iba1, Ionized calcium-binding adapter molecule 1; IFN-γ, Interferone gamma; IL, Interleukin; I/R, ischemia/reperfusion; iNOS, Inducible nitric oxide synthase; IP-10, interferon-gamma induced protein 10; MDA, Malondialdehyde; MEK, MAPK/ERK Kinase; MMP, Matrix metallopeptidase; mTOR, mammalian Target of Rapamycin; NF-κB, Nuclear factor kappa B; NMDAR, N-Methyl-d-aspartate receptor; NO, Nitric oxide; NOX4, NADPH Oxidase 4; NQO1, NAD(P)H Quinone Dehydrogenase 1; ODG/R, Oxygen glucose deprivation and reperfusion; PCP, Poria cocos polysaccharide; PGE2, Prostaglandin E2; PI3K, Phosphoinositide 3-kinases; PON2, Paraoxonase 2; ROS, Reactive oxygen species; SKP2, S-Phase-kinase-associated-protein; SOD, Superoxide Dismutase; STAT, signal transducer and activator of transcription; S100β, S100 calcium-binding protein B; XOD, xanthine oxidase; TNF-α, Tumor-necrosis-factor-alpha; TXNIP, Thioredoxin-interacting-protein. A Literature search was performed from the databases from inception to February 2022 using MEDLINE, Google Scholar, Cochrane Database, CINHAL, CNKI, and Wanfang Med Online. The keywords “Ginseng Radix, Astragalus Radix, Cistanche deserticola Herba, Atractylodis macrocephalae Rhizoma, Poria cocos, Glycyrrhiza Radix, Rhodiola rosea Radix, Epimedii Herba” AND “neurodegeneration, glutamate excitotoxicity, neuroinflammation, oxidative stress, protein aggregation, mitochondrial dysfunction, axonal transport dysfunction, muscle atrophy, spasticity, and ALS” were used as MeSH terms.

## Abbreviations

ALS: amyotrophic lateral sclerosis
ALS-CHEPLA: ALS-Chinese HErbal PLAster
JWLP: *Ji Wu Li* plaster
PLAP: placebo plaster
ALS-SSITS: amyotrophic lateral sclerosis symptom score in integrative treatment scale
ALSFRS-R: amyotrophic lateral sclerosis rating scale-revised
FVC: forced vital capacity
TCM: Traditional Chinese Medicine
EMG: electromyography
CINHAL: Cumulative Index to Nursing and Allied Health Literature
CNKI: China National Knowledge Infrastructure
MeSH: Medical Subject Headings
HSFA: (Chinese) Health Standards for the Use of Food Additives
CFDA: China Food and Drug Administration.
