# Supplementary material for: Slower progression of amyotrophic lateral sclerosis with external application of a Chinese herbal plaster–The randomized, placebo-controlled triple-blinded ALS-CHEPLA trial
Source: Front Neurol. 2022 Oct 17;13:990802. doi: 10.3389/fneur.2022.990802 (PMC9620479; doi:10.3389/fneur.2022.990802)
Supplement: Supplementary file 1 [file Data_Sheet_1.docx]

**Supplementary Text:**

Quality certificates of the herbal materials

Radix Ginseng was obtained from Longbao Shenrong Co., Ltd. (batch number 20210305, ZL-SMP-108-R06-01, Liaoning, China) as dried rhizome. The identity and purity of the compound were confirmed using the Pharmacopoeia of China (China Pharmacopoeia, 2020). The research laboratory of Shanghai Yaofang Co., Ltd. conducted residue analysis and organic trace analysis (Shanghai, China) (C202103-002-002) and confirmed that the heavy metal, pesticide and microbiological contamination was within the guidelines of the Chinese Pharmacopoeia (China Pharmacopoeia, 2020). Radix Astragali was obtained from Shanghai Kangqiao TCM Decoction Pieces Co., Ltd. (batch number 210804, No. C2021-08-0037, Gansu, China) as dried rhizome. The identity and purity of the compound were confirmed according to the Pharmacopoeia of China (Part I, Pharmacopoeia of the People’s Republic of China, 2020, Enterprise internal control standards). The research laboratory of Shanghai Yaofang Co., Ltd. conducted residue analysis and organic trace analysis (FL-03-002-00) and confirmed that the heavy metal, pesticide and microbiological contamination were within the guidelines of the Chinese Pharmacopoeia (China Pharmacopoeia, 2020). Radix Herba Cistanchis was obtained from Shanghai Kangqiao TCM Decoction Pieces Co., Ltd. (batch number 210202, No. C2021-02-0026, Neimenggu, China) as dried rhizome. The identity and purity of the compound were confirmed according to the Pharmacopoeia of Shanghai, China (Shanghai TCM decoction pieces processing specification, 2018, Shanghai, China). The research laboratory of Shanghai Yaofang Co., Ltd. conducted residue analysis and organic trace analysis (Shanghai, China) (FL-03-002-00) and confirmed that the heavy metal, pesticide and microbiological contamination was within the guidelines of the Pharmacopoeia of Shanghai, China (Shanghai TCM decoction pieces processing specification, 2018, Shanghai, China). Rhizoma Atractylodis Macrocephalae was obtained from Shanghai Kangqiao TCM Decoction Pieces Co., Ltd. (batch number 210622, No. C2021-07-0009, Zhejiang, China) as dried rhizome. The identity and purity of the compound were confirmed according to the Pharmacopoeia of Shanghai, China (Shanghai TCM decoction pieces processing specification, 2018, Shanghai, China). The research laboratory of Shanghai Yaofang Co., Ltd. conducted residue analysis and organic trace analysis (Shanghai, China) (FL-03-002-00) and confirmed that the heavy metal, pesticide and microbiological contamination was within the guidelines of the Pharmacopoeia of Shanghai, China (Shanghai TCM decoction pieces processing specification, 2018, Shanghai, China). Poria cocos was obtained from Shanghai Kangqiao TCM Decoction Pieces Co., Ltd. (batch number 210710, No. C2021-07-0071, Anhui, China) as dried rhizome. The identity and purity of the compound were confirmed according to the Pharmacopoeia of Shanghai, China (Shanghai TCM decoction pieces processing specification, 2018, Shanghai, China). The research laboratory of Shanghai Yaofang Co., Ltd. conducted residue analysis and organic trace analysis (Shanghai, China) (FL-03-002-00) and confirmed that the heavy metal, pesticide and microbiological contamination was within the guidelines of the Pharmacopoeia of Shanghai, China (Shanghai TCM decoction pieces processing specification, 2018, Shanghai, China). Radix Glycyrrhizae was obtained from Shanghai Kangqiao TCM Decoction Pieces Co., Ltd. (batch number 210624, No. C2021-06-0165, Xinjiang, China) as dried rhizome. The identity and purity of the compound were confirmed according to the Pharmacopoeia of Shanghai, China (Shanghai TCM decoction pieces processing specification, 2018, Shanghai, China). The research laboratory of Shanghai Yaofang Co., Ltd. conducted residue analysis and organic trace analysis (Shanghai, China) (FL-03-002-00) and confirmed that the heavy metal, pesticide and microbiological contamination was within the guidelines of the Pharmacopoeia of Shanghai, China (Shanghai TCM decoction pieces processing specification, 2018, Shanghai, China). Rhodiola Rosea was obtained from Shanghai Kangqiao TCM Decoction Pieces Co., Ltd. (batch number 210524, No. C2021-05-0157, Xizang, China) as dried rhizome. The identity and purity of the compound were confirmed according to the Pharmacopoeia of China (Part I, Pharmacopoeia of the People’s Republic of China, 2020. Enterprise internal control standards). The research laboratory of Shanghai Yaofang Co., Ltd. conducted residue analysis and organic trace analysis (Shanghai, China) and confirmed that the heavy metal, pesticide and microbiological contamination was within the guidelines of the Chinese Pharmacopoeia (China Pharmacopoeia, 2020).
