# Supplementary figures and images for: Slower progression of amyotrophic lateral sclerosis with external application of a Chinese herbal plaster–The randomized, placebo-controlled triple-blinded ALS-CHEPLA trial
Source: Front Neurol. 2022 Oct 17;13:990802. doi: 10.3389/fneur.2022.990802 (PMC9620479; doi:10.3389/fneur.2022.990802)

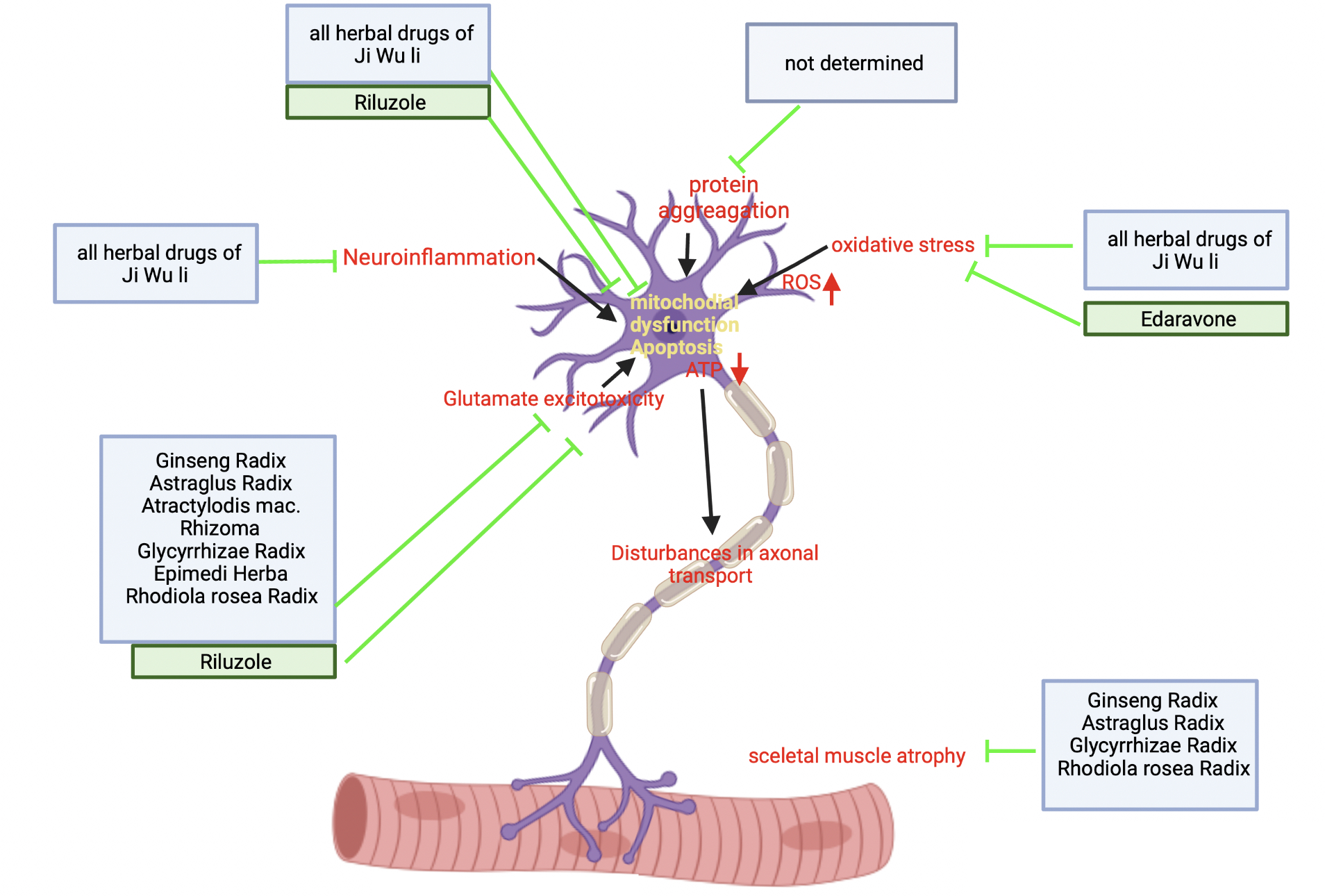

Supplement: Supplementary Figure 1 — Pharmacological treatment targets in relation to ALS disease mechanism. [file Image_1.jpeg]
