# Supplementary material for: Slower progression of amyotrophic lateral sclerosis with external application of a Chinese herbal plaster–The randomized, placebo-controlled triple-blinded ALS-CHEPLA trial
Source: Front Neurol. 2022 Oct 17;13:990802. doi: 10.3389/fneur.2022.990802 (PMC9620479; doi:10.3389/fneur.2022.990802)
Supplement: Supplementary Table 1a — Antioxidative and anti-inflammatory activities of the main constituents of Ji Wu Li. [file Table_1.docx]

Supplementary Table 1a

Antioxidative and anti-inflammatory activities of the main constituents of *Ji Wu Li*
