# Supplementary material for: Slower progression of amyotrophic lateral sclerosis with external application of a Chinese herbal plaster–The randomized, placebo-controlled triple-blinded ALS-CHEPLA trial
Source: Front Neurol. 2022 Oct 17;13:990802. doi: 10.3389/fneur.2022.990802 (PMC9620479; doi:10.3389/fneur.2022.990802)
Supplement: Supplementary Table 1b — Neuroprotective and Glutamate excitotoxicity- and muscle atrophy attenuating effects of Ji Wu Li constituents. ABTS, 2,2′-Azino-bis(3-Ethylbenzothiazoline-6-Sulfonic Acid); AChE, Acetylcholinesterase; AIF, apoptosis-inducing factor; AMPK, AMP-activated protein kinase; Bax, Bcl-2-associated X protein; Bcl-2, B-cell lymphoma 2; CARM1, co-activator-associated arginine methyltransferase 1; CAT, Catalase; COX2, Cyclooxygenase-2; DAPK1, Death Associated Protein Kinase 1; DNA, deoxyribonucleic acid; DPPH, 2,2-Diphenyl-1-picrylhydrazyl; ERK, Extracellular-signal Regulated Kinases; GLT-1, Glutamate transporter-1; GPx, Glutathione peroxidase; GSH, Glutathion in its reduced form; GSH-PX, Glutathionperoxidase; GSK3β, Glycogen synthase kinase 3 beta; HSP-16.2, Heat-shock-protein 16.2; Iba1, Ionized calcium-binding adapter molecule 1; IFN-γ, Interferone gamma; IL, Interleukin; I/R, ischemia/reperfusion; iNOS, Inducible nitric oxide synthase; IP-10, interferon-gamma induced protein 10; MDA, Malondialdehyde; MEK, MAPK/ERK Kinase; MMP, Matrix metallopeptidase; mTOR, mammalian Target of Rapamycin; NF-κB, Nuclear factor kappa B; NMDAR, N-Methyl-d-aspartate receptor; NO, Nitric oxide; NOX4, NADPH Oxidase 4; NQO1, NAD(P)H Quinone Dehydrogenase 1; ODG/R, Oxygen glucose deprivation and reperfusion; PCP, Poria cocos polysaccharide; PGE2, Prostaglandin E2; PI3K, Phosphoinositide 3-kinases; PON2, Paraoxonase 2; ROS, Reactive oxygen species; SKP2, S-Phase-kinase-associated-protein; SOD, Superoxide Dismutase; STAT, signal transducer and activator of transcription; S100β, S100 calcium-binding protein B; XOD, xanthine oxidase; TNF-α, Tumor-necrosis-factor-alpha; TXNIP, Thioredoxin-interacting-protein. A Literature search was performed from the databases from inception to February 2022 using MEDLINE, Google Scholar, Cochrane Database, CINHAL, CNKI, and Wanfang Med Online. The keywords “Ginseng Radix, Astragalus Radix, Cistanche deserticola Herba, Atractylodis macrocephalae Rhizoma, Poria cocos, G [file Table_2.docx]

Supplementary Table 1b

Neuroprotective and Glutamate excitotoxicity- and muscle atrophy attenuating effects of *Ji Wu Li* constituents

*Abbreviations:*ABTS, 2,2′-Azino-bis(3-Ethylbenzothiazoline-6-Sulfonic Acid); AChE, Acetylcholinesterase; AIF, apoptosis-inducing factor; AMPK, AMP-activated protein kinase; Bax, Bcl-2-associated X protein; Bcl-2, B-cell lymphoma 2; CARM1, co-activator-associated arginine methyltransferase 1; CAT, Catalase; COX2, Cyclooxygenase-2; DAPK1, Death Associated Protein Kinase 1; DNA, deoxyribonucleic acid; DPPH, 2,2-Diphenyl-1-picrylhydrazyl; ERK, Extracellular-signal Regulated Kinases; GLT-1, Glutamate transporter-1; GPx, Glutathione peroxidase; GSH, Glutathion in its reduced form; GSH-PX, Glutathionperoxidase; GSK3β, Glycogen synthase kinase 3 beta; HSP-16.2, Heat-shock-protein 16.2; Iba1, Ionized calcium-binding adapter molecule 1; IFN-γ, Interferone gamma; IL, Interleukin; I/R, ischemia/reperfusion; iNOS, Inducible nitric oxide synthase; IP-10, interferon-gamma induced protein 10; MDA, Malondialdehyde; MEK, MAPK/ERK Kinase; MMP, Matrix metallopeptidase; mTOR, mammalian Target of Rapamycin; NF-κB, Nuclear factor kappa B; NMDAR, N-Methyl-d-aspartate receptor; NO, Nitric oxide; NOX4, NADPH Oxidase 4; NQO1, NAD(P)H Quinone Dehydrogenase 1; ODG/R, Oxygen glucose deprivation and reperfusion; PCP, Poria cocos polysaccharide; PGE2, Prostaglandin E2; PI3K, Phosphoinositide 3-kinases; PON2, Paraoxonase 2;

ROS, Reactive oxygen species; SKP2, S-Phase-kinase-associated-protein; SOD, Superoxide Dismutase; STAT, signal transducer and activator of transcription; S100β, S100 calcium-binding protein B; XOD, xanthine oxidase; TNF-α, Tumor-necrosis-factor-alpha TXNIP, Thioredoxin-interacting-protein.

A Literature search was performed from the databases from inception to February 2022 using MEDLINE, Google Scholar, Cochrane Database, CINHAL, CNKI, and Wanfang Med Online. The keywords “Ginseng Radix, Astragalus Radix, Cistanche deserticola Herba, Atractylodis macrocephalae Rhizoma, Poria cocos, Glycyrrhiza Radix, Rhodiola rosea Radix, Epimedii Herba” AND “neurodegeneration, glutamate excitotoxicity, neuroinflammation, oxidative stress, protein aggregation, mitochondrial dysfunction, axonal transport dysfunction, muscle atrophy, spasticity, ALS” were used as MeSH terms.
